# WORKING WITH COMMUNITY HEALTH WORKERS AS ‘VOLUNTEERS’ IN A VACCINE TRIAL: PRACTICAL AND ETHICAL EXPERIENCES AND IMPLICATIONS

**DOI:** 10.1111/dewb.12015

**Published:** 2013-03-22

**Authors:** Vibian Angwenyi, Dorcas Kamuya, Dorothy Mwachiro, Vicki Marsh, Patricia Njuguna, Sassy Molyneux

**Keywords:** developing world bioethics, research ethics, informed consent, clinical trials, sub-Saharan Africa

## Abstract

Community engagement is increasingly emphasized in biomedical research, as a right in itself, and to strengthen ethical practice. We draw on interviews and observations to consider the practical and ethical implications of involving Community Health Workers (CHWs) as part of a community engagement strategy for a vaccine trial on the Kenyan Coast. CHWs were initially engaged as an important network to be informed about the trial. However over time, and in response to community advice, they became involved in trial information sharing and identifying potential participants; thereby taking on roles that overlapped with those of employed fieldworkers (FWs). While CHWs involvement was generally perceived as positive and appreciated, there were challenges in their relations with FWs and other community members, partly related to levels and forms of remuneration. Specifically, payment of CHWs was not as high as for FWs and was based on ‘performance’. This extrinsic motivation had the potential to crowd out CHWs intrinsic motivation to perform their pre-existing community roles. CHWs remuneration potentially also contributed to CHWs distorting trial information to encourage community members to participate; and to researchers encouraging CHWs to utilize their social connections and status to increase the numbers of people who attended information giving sessions. Individual consent processes were protected in this trial through final information sharing and consent being conducted by trained clinical staff who were not embedded in study communities. However, our experiences suggest that roles and remuneration of all front line staff and volunteers involved in trials need careful consideration from the outset, and monitoring and discussion over time.

## Background

Community engagement is increasingly emphasized as central to biomedical research in international settings, both as a right in itself, and as a means to uphold ethical principles, enhance protection and benefits, create legitimacy, share responsibility between researchers and communities, and strengthen science.^1^ Communities can potentially be involved in a broad range of research activities, from protocol development, to research conduct, reviewing access to data and samples, and dissemination or publication of research findings. Community members are also often employed in research studies to simultaneously recruit, and conduct research processes such as interviews and simple study procedures. Less commonly community members may also recruit participants as part of Peer Driven Recruitment (PDR) or community-based participatory research.^2^

There has been relatively little published information about the experience with community engagement in low income settings, including information about the ethical issues and dilemmas associated with who the ‘communities’ are, and who is selected by whom to represent those communities in what way.^3^ In this paper we share our experiences of having included community health workers (CHWs) in community engagement activities for a vaccine trial, as encouraged by members of the Ministry of Health in Kilifi, Kenya. Following an overview of relevant literature and some background to the trial site, we describe the shifting role of CHWs within the trial over time, from initially being considered a key community to inform, to increasingly involving them in information giving to community members about the trial, and identification of potential trial participants. We consider the practical and ethical implications – both positive and challenging – of having CHWs and employed fieldworkers (FWs) working at the interface with community members, with overlapping roles, and of CHWs’ involvement essentially developing into a form of peer recruitment. We show that the type and level of CHW remuneration and support, and how this differed to that of FWs, contributed to some relationship challenges and potentially to some distortion of trial information by CHWs, and how the possible negative implications were minimised.

### Engaging communities in trials

Communities can be defined based on geography, on special interests or goals, or on shared situations or experiences, with key communities relevant for research likely to include health care system and research staff, as well as the general public and potential research participants.^4^ Available information suggests that researchers often interact with both existing structures within communities of interest (for example, chiefs and community leaders, leaders of women's groups or health support groups, and health care facility committees), and with structures that have been specifically established, with the most widely cited example of the latter being Community Advisory Boards (CABs), or variants of these.^5^ While working with these channels can strengthen research relationships and ethical practice, documented challenges, particularly of working with specifically established structures, include: ensuring clarity in roles and forms of representation; facilitating appropriate selection of members; balancing motivation of members against the need to ensure adequate independence from researchers in a way that facilitates critical and meaningful dialogue; and avoiding politicisation.

With regards to the specific issue of involvement of community members in recruitment, a range of ethical and practical strengths and challenges are recognised.^6^ Potential strengths include remuneration for those employed, enhanced research through improved access and responsiveness to local communities, and strengthened consent processes that encourage potential participants to feel greater comfort and ease to ask questions and understand information and its’ implications. Ethical challenges potentially include exploitation of local recruiters through unfair employment practices, recruiters exploiting the trust of peers in their efforts to meet recruitment quotas (including through compromising consent processes), and privacy and confidentiality breaches.^7^ The latter two concerns feature particularly where community members have prior relationships with potential participants and in cases where recruiters are paid according to performance measures. In addition to these vertical forms of exploitation (wherein a group of outside researchers exploits the social connections that recruiters have with members of the local community), there are also potentially horizontal forms of exploitation where ‘select members of the local community recognise the potential to partner with outside researchers in a way that allows them to gain power and influence within their community’.^8^

Such challenges suggest that the manner in which community members are recruited should be carefully considered as part of a broader framework of community engagement, and that this framework should include a broad range of communication channels or sets of representatives. For health research, the Ministry of Health (MoH) is likely to be an important ‘community’ to include in community engagement plans: health research is often conducted within or linked to health care facilities; community members may often consult health care staff or managers about studies being conducted in their communities; and research activities have the potential to support or undermine health care systems in the short and longer term.^9^

### CHWs a relevant trial community

At the local community level, one potential channel linked to the health care system is Community Health Workers (CHWs). CHWs are “selected by community members, trained to carry out one or more health care functions, answerable to communities for their activities and supported by the health care system.”^10^ They have reportedly played an important role in health care in many developing country settings: by filling in service provision gaps where more skilled personnel are not available; by broadening health care access and coverage in remote areas; by helping attain millennium development goals such as childhood immunization; and by serving as a bridge between professional health care staff and communities.^11^ However CHWs have also faced numerous challenges, including unclear roles, inadequate or inappropriate incentives, unmet training needs and supervision, high attrition rates, and lack of (social) recognition.^12^ Including CHWs in research trials can potentially build on the strengths of such a network, and contribute to overcoming some of their challenges, including strengthening their motivation and recognition. In research, CHWs have been drawn upon to assist researchers to access and educate targeted populations, to act as data collectors, and to help recruit potential participants and conduct reminder visits.^13^ However there is little published documentation about the experiences of involving this key group in research.

In Kenya, CHWs, referred to locally as ‘*madaktari wa vijijini*’ (village doctors), are recruited and trained by the MoH. CHWs have been promoted to the public as key players in the health care system since the 1970s and early 1980s, as part of broader national efforts to strengthen primary health care, and have faced similar achievements and challenges to those in other settings.^14^ They are expected to play an increasingly central role at the interface between communities and health care systems with the roll out of the national ‘Community Strategy’, in which a large network of CHWs are identified and trained to link households to governing structures at location, sub-locational, village, and health facility levels. Within the community strategy, CHWs roles include delivering health care messages, collecting health-related data, and relaying information and referring sick people to health care facilities.

### The trial and trial setting

The vaccine trial site of interest is in Kilifi District, on the Kenyan Coast, led by the KEMRI-Wellcome Trust Research Programme.^15^ Within the research Programme, a core group of community facilitators, the Community Liaison Group (CLG) coordinates Programme-wide and study-specific community engagement activities.

The vaccine trial was set up in three rural health care facilities in Kilifi district, enrolling children aged less than 17 months old from villages surrounding the health care facilities. Following a complete health check-up, participants were randomized into one of three research trial arms to receive either the vaccine under investigation or a comparator vaccine. Fieldworkers from the local community were employed to assist with informing community members about the trial, and identifying potential participants. They also conducted home visits for six consecutive days after vaccination and once a month over the three year study period to check on participants’ overall health. Benefits for participants included free treatment and transport to health care facilities, and 24 hour medical services at local public health care facilities over the entire study period.

For all studies involving participants in Kilifi, study teams are requested, with the support of the CLG, to consider whether or not a community engagement strategy is needed, and if so, what key issues need to be discussed and addressed when and with whom. As with many local research studies,^16^ the vaccine trial strategy included interactions with a range of communities and individuals, including Ministry of Health (MoH) managers (see Box 1).

**Box 1. Summary of community engagement activities**

**Table:** 

PERIOD	ACTIVITY	WHO INVOLVED
Month 1	Consultation and sensitization of Kilifi District stakeholders	*1. MoH structure:* District Medical of Health and District Health Management Team at Kilifi District Hospital. All health facility in-charges working in Kilifi District.
		
		*2. Provincial administration structures:* District Commissioner, Senior District Officer, all chiefs working in Kilifi District

Months 2–6	Community entry and sensitization of stakeholders in Sites A, B and C respectively	*1. MoH Structure:* Dispensary health committees, dispensary staff (facility in-charges, nurses, public health officers, community health extension workers), and community health workers (CHWs)
		
		*2. Local administration:* District officers, local (assistant) chiefs and village elders
		
		*3. Others:* Primary school head teachers, religious leaders, Vitengeni District Stakeholders Forum

Months 8–13	Identification and recruitment of 5–17 month old children (N = 600)	CHWs and fieldworkers

Months 15–27	Identification and recruitment of 6-12 weeks-old children (N = 304)	CHWs and fieldworkers

From month 8	Follow up of research participants	Fieldworkers
	
	Continuous feedback to and from community	Fieldworkers and other key gatekeepers.
	
	Feedback of results	Involves all of the above e.g. Preliminary study results disseminated

## Methods

We conducted a multi-method social science study alongside the trial, including observations of community engagement and consent processes, and interviews with all key stakeholders. In this paper we draw upon in-depth interviews (IDIs) with parents who were approached to enrol their children in the trial (n = 25), three of whom were CHWs, on a household survey with parents of participants (n = 200), on IDIs with staff involved in designing and implementing the community engagement plan (n = 5) and on group discussions with study fieldworkers (n = 3). These interview data supplement semi-structured observations of numerous community engagement activities in homes and health facilities. Interviews and observations were conducted by Vibian Angwenyi and four trained senior fieldworkers employed within CLG.

IDIs were tape recorded, transcribed, translated into English (where necessary), and managed using NVivo 8.0. Two researchers (Vibian Angwenyi and Sassy Molyneux) independently identified emerging themes for analysis. The study protocol was approved nationally, and advanced informed consent was obtained from all interviewees.

## Findings

### Why involve CHWs in trial community engagement, and defining their roles

The trial community engagement strategy is summarised in Box 1. Beyond the initial district level discussions, the trial and associated community engagement activities were implemented in each of the three sites in turn (i.e. the health care facilities and surrounding villages): site A, followed by site B and then site C.

CHWs were initially included in the community engagement plan as a group to be informed about the study in the areas in which the trial would be conducted, but over time they became more involved in information giving and ‘mobilization’ (i.e. the identification of potential study participants in the community and referring them to the trial team for more detailed study information and for consent processes). This greater role was initiated in early discussions with District Health Management Team members, who were keen that the trial be integrated into the national community strategy roll-out:

R1: … the MoH recommended that we use the CHWs because they are at the grassroots, we don't need to put some other new people in, we didn't need something like a CAB to do that activity. So he came out strongly on that. (IDI02_Staff)

A challenge experienced from the outset with CHWs involvement, and constantly re-negotiated, was their precise role in the trial. Different stakeholders involved in the community engagement plan differed in their views. For example, MoH official(s) initially wanted CHWs restricted to identifying potential participants, while local leaders and some research staff were keen that CHWs were also involved in active information sharing about the trial and referring potential participants to the trial team. As a staff member explained:

R1: … I did feel that we should use them [CHWs] to enrol but the [MoH officials] felt that we shouldn't really give them a job as such…so [site A] said ‘why don't we allow the CHWs to mobilize’, that helped. When we went to [site C] when we had a meeting with the chief, village elders and CHWs they said “well we have CHWs they'll mobilize …” (IDI04_staff)

In site A, CHW roles were initially restricted to visiting people at home with fieldworkers. During those periods of time when recruitment was difficult, CHWs also assisted through home visits with research staff to enquire about reasons for refusal and help clear any misconceptions about the trial. In site B and C, CHWs from the outset were more centrally involved as primary mobilizers: informing potential participants about the trial and inviting them to information and consent sessions by trial team members at the health care facilities.

### CHWs-fieldworker relationships and interactions

Shifting CHWs roles over time was linked in part to the number of fieldworkers in the trial, and the relationship between CHWs and fieldworkers. In KEMRI-Wellcome Trust, ‘fieldworkers’ are frontline staff, employed by KEMRI from the local communities in which study participants reside, to undertake specific study related activities.^17^ Employment is a high priority for local communities, given the low income and high unemployment levels in the area. In community engagement activities for the broader programme, community members have often therefore argued for more transparent employment procedures, and more evenly spread employment across the communities where much of the programme's research is conducted (i.e. the 250,000 people living in the main district hospital catchment area). One approach adopted to respond to these requests has been to, wherever possible, employ fieldworkers from the villages in which the research will take place.^18^ For this trial, this approach meant that both FWs and CHWs often came from the same communities.

FWs receive a salary and undergo specific training in topics such as research ethics and informed consent, as part of the trial's recruitment protocol, whereas for this trial CHWs were unpaid ‘volunteers’ who received no formal training in their research-related activities beyond being issued with simple messages and study leaflets. CHWs were given some compensation for their role in the trial. Rate of compensation was initially based on MoH guidelines (approximately $2.50 per day), but over time compensation was linked on the basis of advice from local stakeholders to performance i.e. the number of participants a CHW was able to encourage to visit the health care facility to hear more about the trial from trial clinicians. When compensated by performance, CHWs were given about $2.50 for four potential participant parents. In site A, an initial activity by CHWs of registering all names of children eligible by age was also compensated for, at a rate suggested and agreed with the Health Facility Committee, which includes the health care facility in-charge and elected community representatives.

In site A, by the time CHWs were involved in the study, FWs were already employed and trained. There were therefore relatively clear demarcations in roles between FWs and CHWs. In the other two sites CHWs were more heavily involved in identifying potential participants from the outset of the study, partly because FWs were hired and trained after participant recruitment had started. Over time, in all the three sites, CHWs became less directly involved in the study in relation to FWs:

R1: … their roles are different [FWs and CHWs] and we explain to them. The roles of … CHWs end at the point where they have invited eligible study participants to the dispensary… they don't have any direct contact with study participants, they will just be helping them [FWs] as … leaders of the community [as they go about their] normal activities … in case they come across an issue, then they would make an effort of seeing the field worker … (IDI03_staff)

Overall, the relationship between CHWs and FWs appeared to be mutually supportive. However there were some challenges. In group discussions with FWs, for example, some CHWs reportedly distorted study information causing difficulties for FWs in recruitment, or would only assist FWs in mobilization if they were compensated. The latter was linked to apparently overlapping activities but with different levels of remuneration; a challenge understood by FWs:

S21: So as we went round he [CHW] used to say “you my colleagues earn but for me I go round and get nothing. You have bicycles and we have nothing but when we go, we go together. It's like I am helping you in your work yet no one looks after us.” So that is one of the challenges. But honestly if I look at it fairly its true; we climb hills together so you find there is some difficulty in convincing him … if he had gone to work [he would have] earned something for a living … so it becomes hard because he wants something from there and you see I can't help them. At times it can go to an extent of them asking “why can't KEMRI help us out in this work” so I told them I can't answer or promise anything. (FWs group interview 3_site B).

### Overall level of activity and impact on the trial

In IDIs, community members and trial staff reported that CHWs played an important role in the trial. CHWs were considered by trial staff and some community members to be easily accessible and approachable for discussions on study-related issues: they are relatively well-known and mature (older) when compared to FWs; and the nature of their tasks fitted well with their broader CHW roles (Box 2: quotes 1–3). Survey data supported that a third of participants’ parents (n = 63; 32%) had heard about the trial from a CHW before they joined, with a quarter reporting that CHWs remained important informants. Most interviewees (n = 169; 91%) recommended that CHWs specifically be informed in future research.

**Box 2. Strengths and challenges of working with CHWs in the trial (illustrative quotes)**

Quote 1: ‘… those [CHWs] were the people who could actually mobilize [help identify] people to come and join the study coz they are used to giving out sort of health messages … the initial mobilization [identification of study participants] would be heavily assisted by the CHWs because they have direct contact, they are a bit more mature and they probably have more community standing than a young fieldworker’. (IDI04_Staff)

Quote 2: ‘Yes [our roles as CHWs] was of importance. If you explain to people and they agree to go with their kids and get treated, they must appreciate you, they will say “had it not been this person my child wouldn't have been getting this treatment”. So you are assisting the community’. (IDI14_female parent/CHW, site C)

Quote 3: ‘… there was some scepticism about using the community strategy and even I was a bit sceptic about it because this is not something we have done. But seeing that this is a new area and there is nothing [i.e. no other formal mechanism for the area such as CABs or KCR] … am quite impressed that using the community strategy it seems to somehow work’. (IDI01_Staff)

Quote 4: ‘She tried to explain more to me and she thought that I had not understood about the study. Therefore she came to me again and explained again very well and I absolutely understood her but then the decision is, I had already decided.’ (IDI21_male parent, site A)

Quote 5: ‘… we were stopped from entering one home, we did that three times, we were told “go back because we already know what brings you here”. The third time we went to see if these people had changed their thoughts and they told us, “it's like you have nothing to do, why do you all the time go round to homes looking for children to be enrolled in the vaccine. Does it mean people were not treated before KEMRI came?” It was very discouraging. We just had to endure but how they were talking was not a nice experience at all.’ (IDI09_female parent/CHW, site C)

However there were some challenges associated with CHWs activities, linked specifically to their role in the trial. These challenges included a perception by some trial staff and participants that CHWs were over-emphasising study benefits during information giving, and a corresponding perception by some participants that CHWs were trying to exert some form of pressure on them to participate. In their efforts to encourage people to learn more about the trial, CHWs sometimes faced hostility, especially from community members who were not keen to participate or at other times when community members expected CHWs to enrol their own eligible children, as a precondition for their enrolment (Box 2; quotes 4 and 5). Furthermore, it is possible that working for the trial was undermining CHWs’ normal day-to-day activities, or at least some parents’ views of how well they were performing their roles:

R1: You know in the beginning they were really concerned with the health of children but since KEMRI came it's mostly FWs who come to visit these children at home … CHWs take a long period of time before they come. In fact since KEMRI activities started we haven't seen them. Not unless you have questions then you follow them. (IDI12_female parent, site C)

### Key factors influencing CHWs perceived effectiveness in supporting the trial

#### 1. Prior functioning of CHWs, and prior exposure to KEMRI and the trial

There were differences in CHWs structure and organisation across the three sites before the trial was introduced, and differences in prior exposure to KEMRI. In site C, where trial staff felt performance of CHWs was particularly impressive, CHWs organised and held regular meetings among themselves before the trial, had participated in previous trainings and workshops organised by non-governmental organisations (NGO) or the MoH, and had interacted with KEMRI in a previous study. In sites A and B, during trial recruitment CHWs were newly recruited and trained under the community strategy, and this group was already experiencing a high rate of attrition. They also had little or no prior exposure to KEMRI and expressed some scepticism about the trial at the outset:

R1: … in site C the response was so good they [CHWs] were all excited about the study and they were willing to help … but in site A, I think the CHWs themselves were not enthusiastic about the study so they didn't receive it with a lot of weight. That's why they were a bit reluctant; some of them were active but majority were a bit reluctant to work with us. (IDI03_Staff)

#### 2. Incentives

Compensation was considered crucial to CHWs motivation in this and all other community activities they are involved in, but it was also associated with some pragmatic challenges around exactly how a fair compensation system would be established:

R1: … In site A because we didn't actually use them [CHWs] to mobilize we didn't have a standard way of doing it. So when they were collecting names we assumed that you would do it in so many days, and therefore paid them for so many days. But it brought a bit of confusion and concerns because some people said “well I walked round and saw all my children and so and so just sat down and wrote them from their head …” (IDI04_staff)

The performance model in particular was considered very motivating to CHWs. However it also clearly resulted in competition and struggle among CHWs:

R1: … we were told if you get many mothers you will get a ‘big gift’ … by that time every CHW was struggling to get mothers. You would find about 4 CHWs going to one homestead … you'd hear the CHWs saying “even me I went there and advised them” … we therefore have to divide that amount because everyone claims they went to advise … (IDI09_female parent/CHW, site C)

Negotiations for increased allowances remained a feature in CHWs meetings with trial staff in all sites, including site C.

#### 3. Other support for CHWs from KEMRI and the community

CHWs were trained by the trial team to conduct their roles. However given that they were not expected to discuss the trial in detail with potential participants, or consent participants, their training was necessarily less in-depth than that of paid full time staff, including field workers. CHWs interviewed felt that they were ill-equipped relative to fieldworkers with study information, which in turn limited their ability to address community concerns. They also expressed a desire to have more frequent meetings with senior study staff over the course of the trial, and not just during busy times such as recruitment.

During KEMRI-CHWs meetings we observed that in addition to requests for more allowances (noted above) there were also demands from CHWs for bicycles (site B), and frequent requests for employment particularly when FW positions were being advertised:

R1: Can you [KEMRI] employ people if they have such a certificate [i.e. a CHW training certificate]? … we are asking this because sometimes we have certificates which are just lying idle in our houses and if there are any chances we can also apply. We have been volunteering since 2000 up to now … (IDI14_female parent/CHW, site C)

Although the positive perception of CHWs in the local community was described above, there was also some hint that they were not always considered to be knowledgeable or active:

R1: Yes, CHWs can be (pause) those are not CHWs but volunteers. They carry out these roles but not very keenly because they know nothing. They are like ‘Red Cross’. So you will find their mobilization is not so good … (IDI08_male parent/CHW, site A)

## Discussion

Representatives of the health care system can be important players to include in community engagement strategies in clinical trials. CHWs are clearly an important group to consider interacting within community- based trials given their position at the interface between health care systems and local communities. Furthermore, as in our setting, engagement with CHWs is likely to be recommended by community members and representatives. Beyond simply being informed of research as part of community sensitisation activities, CHWs can be given the more proactive roles they had in this trial, such as introducing trial team members to community members, assisting the trial team with identification of potential participants, sharing information about the trial, and responding to trial- related concerns in the community.

Echoing some of the debates expressed in the wider literature, our experience suggests that having CHWs work with other frontline staff performing similar roles has potential practical and ethical benefits and challenges. Studies benefit from working with and learning from well-known and respected individuals in the study communities, who in turn appreciate being given financial support (where this is given) to conduct activities that appear to be particularly related to their training and that increase their visibility locally. As discussions nationally continue on what support CHWs should be given by the Ministry of Health to roll out the community strategy, and where the funds to support this should come from,^19^ CHWs are expected to continue performing their roles either on an entirely voluntary basis, or with the support of locally active governmental or non-governmental organizations. Offering some compensation for CHWs to be involved in research-related activities, as was done for this trial, appeared to assist in keeping CHWs motivated and active over the course of the trial, as was envisioned by MoH staff and community elders who recommended their involvement. The importance of ensuring that there is adequate motivation for CHWs, whether it is extrinsic or intrinsic, and financial or non-financial, is widely recognised internationally.^20^ It is feasible that the financial contribution offered by the trial to CHWs in our context assisted in some small way the implementation of the national community strategy in the trial communities. Involvement of CHWs in community engagement was also perceived by trial staff to assist indirectly with ensuring that expected numbers of participants were recruited into the trial through encouraging potential participants to come and hear about the study, and to strengthen relationships and trust more broadly between the trial team and the communities in which the research was being conducted. Given that strengthened science and appropriate levels of trust are often included as ethical goals of community engagement strategies,^21^ engagement of CHWs in the ways described in this paper, could be described as a potentially important element of a wider set of community engagement activities. Although the roles of CHWs evolved over time, leading to differences across the three sites in the nature of how CHWs were involved, this is to some extent inevitable, given community engagement can never be a pre-fabricated set of activities applied uniformly across all settings, but rather a dynamic and ever changing set of negotiated relationships.^22^

However, there are clearly dilemmas associated with involving CHWs and other frontline research staff. Firstly, although there was a clear distinction maintained between employees and CHW volunteers, with the former having more diverse roles and training (in research, ethics, and trial details), there was some indication of overlaps in roles, of tensions between CHWs and fieldworkers, and of conflicts among CHWs themselves. In particular there were suggestions that CHWs were undermining study-related information provided to potential participants by fieldworkers, and exerting some pressure on participants to visit health care facilities for further information in order to increase their own reimbursements. These findings could be interpreted as indicating both vertical exploitation – where CHWs were being encouraged by researchers to exploit their social connections and status in the community – and horizontal exploitation, through CHWs seeking to gain income and influence in their communities.^23^ However, both of these forms of exploitation were eased by excluding CHWs from any final information giving or consenting for the trial; roles which were performed individually by carefully trained study clinicians. CHWs were also inadequately trained to understand and share key trial messages, and may therefore have been simply ill-informed or unable to give correct information or answers to community members. Furthermore, as community members with a keen interest in health care, CHWs may have been less interested in their own gains than in ensuring that households had the opportunity to access what were indeed significant health related benefits associated with trial participation for participants. Given the high degree of poverty and unemployment levels in the trial communities,^24^ the inadequate resources allocated to support CHWs by Ministries of Health and NGOs in Kenya as elsewhere (noted above), and the significant health-related burdens and costs facing low income households in these communities, these challenges and tensions between interface staff and volunteers are not surprising or unreasonable, including to fieldworkers. These findings suggest the importance from the outset of carefully considering (and discussing and re-considering) the roles, training and support systems of CHWs and how these relate to those of FWs. In so doing, it should be recognised that fieldworkers themselves also face many similar ethical and practical challenges in their roles at the interface, with their level of embeddedness in the particular study communities influencing their familiarity with local social networks and norms, and therefore the way in which they experience and handle these challenges.^25^

A second dilemma associated with including CHWs in the trial, possibly motivated by the hope of remuneration or future employment in the research programme, was some indication that their involvement might have impacted negatively on their pre-existing CHW roles in the community. This was possibly more likely where CHWs were not already highly active and with clear roles and relationships within the community. This would be plausible either through CHWs spending significant amounts of time on trial-related activities, or through undermining their relationship with community members through repeated visits to homes as part of their mobilization efforts. In both cases, extrinsic incentives might also have begun to crowd out CHWs intrinsic motivation such as social recognition, knowledge gain, and the opportunity to make a social contribution.^26^ More broadly therefore, engagement of CHWs in this way can potentially undermine rather than support CHW programmes which are ultimately intended to benefit community health, with the possibility of this depending on pre-existing dynamics of the cadre. Clearly this would operate against the ethical gains of working with CHWs described above, and against community leaders’ initial motivation for suggesting CHWs involvement in the trial. Specifically it would potentially undermine fair benefits in research through reducing benefits to communities during and after trials, and potentially cause harm or disadvantage through undermining community engagement in local health care systems. It was beyond the scope of our study to explore this in depth, or the long term implications of CHW involvement in this trial. However, this finding suggests the need to recognise differences among CHWs, and to consider and monitor such potential perverse outcomes of engaging with CHWs, in future trials, and more specifically of different CHW reimbursement strategies. Depending on the context, careful discussion and agreement with Ministry of Health and NGO managers and implementers is likely to be important.

Another potential challenge in paying some level of remuneration to CHWs, although not identified as a challenge in this study, is that other community leaders and representatives who are informed about a trial and who typically help to raise and respond to community concerns, might also be keen to receive some financial support for any involvement they (perceive themselves) to have in the trial. The dilemma with providing motivation for other community ‘volunteers’ (for example chiefs and elders, and women's group representatives), as for CHWs, is the possibility of crowding out any sense of intrinsic motivation. For all groups, there is also a concern that increasing motivation of community members has to be balanced against the need to ensure that they maintain an independence from researchers in a way that facilitates critical and meaningful dialogue. On the other hand there should be recognition of community members’ contributions, and efforts to minimise trial costs – in time and especially financially – for community members. Challenges in achieving an appropriate balance have been regularly observed for community advisory boards. Where there is no motivation, or independence and dialogue is compromised, the potential of community engagement to strengthen research relationships and ethical practice is undermined. This opens up the possibility to identify other forms of motivation that minimise such limitations, including for example providing appropriate training or exposure to health care research.

## Conclusion

We have identified a range of practical and ethical benefits and challenges of involving CHWs proactively in a community-based vaccine trial. How these benefits and challenges balance out is difficult to fully predict in advance of a study. That the form of involvement of CHWs and how they were motivated to do this shifted over the course of the trial, and continues to do so, is perhaps an inevitable aspect of a broader community engagement activity that is designed to be constantly listening to and responding to issues raised by key local stakeholders. However there are some lessons that emerge from this experience. These include the importance from the outset and over time of carefully considering (and discussing) the roles of CHWs and how these relate to those of FWs and other community representatives, ensuring that there is clarity in those roles for all key players at the local level, and providing adequate training, supervision and financial support for those roles to be performed.
